# Effect of microbial diversity and their functions on soil nutrient cycling in the rhizosphere zone of Dahongpao mother tree and cutting Dahongpao

**DOI:** 10.3389/fpls.2025.1574020

**Published:** 2025-05-08

**Authors:** Xiaoli Jia, Lei Hong, Yulin Wang, Qi Zhang, Yuhua Wang, Miao Jia, Yangxin Luo, Tingting Wang, Jianghua Ye, Haibin Wang

**Affiliations:** ^1^ College of Tea and Food Science, Wuyi University, Wuyishan, China; ^2^ College of Life Science, Longyan University, Longyan, China; ^3^ College of JunCao Science and Ecology, Fujian Agriculture and Forestry University, Fuzhou, China

**Keywords:** tea tree, macrogenomics, microbial diversity and function, soil enzyme, soil nutrient cycling

## Abstract

Dahongpao mother tree (*Camellia sinensis*) is nearly 400 years old and is the symbol of Wuyi rock tea. It is unclear whether the structure and function of the rhizosphere soil microbial community of Dahongpao mother tree (MD) and its cutting Dahongpao (PD) change after planting. In this study, macrogenomics was used to analyze the structure and function of rhizosphere soil microbial communities, as well as to explore their relationship with soil nutrient transformations in MD and PD tea trees. The results showed that pH, total nitrogen, total phosphorus, total potassium, available nitrogen, available phosphorus and available potassium were significantly higher in the rhizosphere soil of MD than in PD by 1.22, 3.24, 5.38, 1.10, 1.52, 4.42 and 1.17 times, respectively. Secondly, soil urease, sucrase, protease, cellulase and catalase activities were also significantly higher in MD than in PD by 1.25-, 2.95-, 1.14-, 1.23-, and 1.30-fold. Macrogenomic analysis showed that rhizosphere soil microbial richness and diversity were higher in MD than in PD. There were eight characteristic microorganisms that significantly differed between MD and PD rhizosphere soils, and the results of functional analysis showed that MD rhizosphere soil microorganisms had higher carbon, nitrogen, and phosphorus biotransformation capacity, were more conducive to the accumulation and release of nutrients in the soil, and were more conducive to the promotion of tea tree growth. The results of PLS-SEM equation analysis showed that characteristic microorganisms positively regulated soil microbial function (1.00**), enzyme activity (0.84*) and nutrient content (0.82*). It can be seen that the abundance of soil characteristic microorganisms in the rhizospehre soil of MD increased significantly compared with that of PD, prompting a significant enhancement of their corresponding functions, which was more conducive to soil improvement, increased soil enzyme activity, enhanced soil nutrient biotransformation, and then increased soil nutrient accumulation and effectiveness, and promoted the growth of tea trees. This study provides an important theoretical basis for microbial regulation of tea tree cuttings management.

## 1 Introduction

Dahongpao mother tree is nearly 400 years old with six plants growing in Jiulongke Scenic Area, Wuyishan city, Fujian province, China (117°57′19.098″ E, 27°40′17.8212″ N) (Chen, 2015). Dahongpao mother tree is the symbol of the tea industry in Wuyishan City, and in 2000, the government of Wuyishan City listed Dahongpao mother tree as a key conservation target. To effectively protect the Dahongpao mother tree, harvesting for tea production has been prohibited since 2006 (Ng et al., 2018). Dahongpao mother tree is so small in number that even if harvested, only a small amount of tea can be made, which is not enough to meet market demand. Therefore, in order to accelerate the popularization and application of Dahongpao tea tree and thus promote the development of the tea industry in Wuyishan City, a large number of Dahongpao tea trees were produced in the mid-1980s by cuttings from Dahongpao mother tree (Hou and Wu, 2019). A large number of Dahongpao finished teas sold in the market today are processed from leaves of tea tree cuttings. This method has played an important role in promoting the sustainable development of the tea industry. However, the large number of cuttings also brings some problems. For example, the growth and quality of Dahongpao tea tree cuttings changed due to differences in the soil environment (Ye et al., 2024), so it is important to explore in depth the differences in rhizosphere soil microenvironments between Dahongpao mother trees and cuttings for cuttings management.

Plant cutting is a fast and efficient method of asexual propagation that ensures that the genome of the cuttings is identical to that of the parent and that they are able to efficiently inherit the basic characteristics of the parent (Smith et al., 2022). However, there are still some differences between cuttings and their parents in tree age, and this difference leads to differences in growth and defense (Hewitt, 2020; Xiao et al., 2023). Soil is the vehicle for planting, and after planting, rhizosphere microbial interactions at the plant-soil interface alter the soil environment and affect plant growth (Zhao et al., 2021). It has been reported that plant cuttings planted in the same plot as their parent plant under the same management practices showed significant changes in the community structure and function of rhizosphere soil microorganisms, which in turn led to plant growth and quality, showing significant differences (Singh, 2021). Wang et al. (2024a) investigated the microbial diversity and function of soil microorganisms in the rhizosphere of cutting *Gastrodia* and found that as the number of asexually propagated generations of *Gastrodia* increased, their rhizosphere soils became progressively less probiotic, while pathogenic bacteria continued to increase, and soil nutrient biotransformation was reduced, and the growth of *Gastrodia* slowed down. Adomako et al. (2022) found that rhizosphere soil microorganisms of asexually propagated *Solidago canadensis* can alter soil nitrogen-phosphorus ratios, leading to soil nutrient imbalance, which in turn affects the productivity of *Solidago canadensis*. Lin et al. (2024) also found that the abundance of beneficial microorganisms in asexually propagated sugarcane rhizosphere soils was significantly reduced, soil enzyme activities were reduced, nutrient conversion was reduced, and sugarcane growth was impeded. It can be seen that although asexually propagated plants can effectively inherit the characteristics of the parent, the community structure and function of their rhizosphere soil microorganisms changed significantly during cultivation, altering the biotransformation of soil nutrients, which in turn affected plant growth.

Tea tree is an economically important plant and is mainly grown from cuttings (Ng et al., 2018). Ye et al. (2024) found that the quality of tea trees from cuttings was significantly lower than the parent, and concluded that cuttings had a significant effect on the growth and quality of tea trees. Jia et al. (2024) found that tea tree growth and quality were significantly correlated with the abundance of soil characteristic microorganisms and their functions, and that characteristic microorganisms could alter nutrient transformations in the soil, which in turn could affect soil available nutrient content and affect tea quality. It can be seen that there are indeed some differences in the growth and quality of tea trees from cuttings and parent tea trees. It is hypothesized that the cause of this difference is a change in the community structure of rhizosphere soil microorganisms between the cuttings and the parent tea tree, which in turn leads to differences in the abundance and function of certain characteristic microorganisms, altering the biotransformation ability of soil nutrients and affecting the growth of the tea tree. Therefore, it is important to explore in depth the effect of characteristic microorganisms and their functions on the biotransformation of soil nutrients that distinguish cuttings from the parent tea tree. Accordingly, in this study, Dahongpao mother tree (MD) and cutting Dahongpao (PD) were used as materials to collect rhizosphere soils of tea trees, and to determine basic physicochemical indexes and soil enzyme activities. At the same time, the microbial community structure of tea tree rhizosphere soil was determined by macrogenomics technology and screened to obtain characteristic microorganisms distinguishing MD and PD, and the effect of characteristic microorganisms and their functions on enzyme activity and nutrient biotransformation capacity of tea tree rhizosphere soil was analyzed. The results provide an important theoretical basis for microbial regulation of tea tree cuttings management.

## 2 Materials and methods

### 2.1 Sample sampling

This study was conducted on Dahongpao mother tree (MD) and the first asexually propagated cuttings of Dahongpao (PD) in the 1980s. Both MD and PD were planted in Jiulongke Scenic Area, Wuyi Mountain, Fujian Province, China (117°57′19.098″ E, 27°40′17.8212″ N), and the ages of the trees were about 390 and 40 years, respectively. MD and PD were planted in the same area with a straight line distance of about 20 m between them. The soil of the planting site is red gravelly rock, with an average annual temperature of about 12°C to 13°C, an annual precipitation of more than 2000 mm, and a relative humidity of up to 85%. In May 2023, rhizosphere soils of MD and PD tea trees were collected for soil basic physicochemical indexes, enzyme activities, and macrogenomic sequencing analysis, with three independent replicates for each sample. Rhizosphere soil was sampled by randomly selecting two tea trees for each sample, removing dead leaves and residues on the soil surface, shoveling the surface soil layer by layer for about 20 cm until the root system of the tea tree appeared, and collecting the soil attached to the root system of the tea tree and mixing it thoroughly, which was a repetitive sample of the rhizosphere soil of the tea tree (Wang et al., 2024c).

### 2.2 Determination of basic physicochemical indexes of soil

In this study, the basic physicochemical indexes of tea tree rhizosphere soil were mainly determined as soil pH, organic matter content, total nitrogen, total phosphorus, total potassium, available nitrogen, available phosphorus and available potassium, and specific methods were referred to Wang et al. (2023a). The brief description is as follows: pH was determined by water extraction potentiometry; organic matter content was determined by potassium dichromate oxidation and sulfuric acid titration; total nitrogen was determined by sulfuric acid high-temperature cooking and Kjeldahl nitrogen determination; available nitrogen was determined by NaOH solution extraction and hydrochloric acid titration; total phosphorus was determined by NaOH melting and molybdenum-antimony antimetric colorimetry; available phosphorus was extracted by NaHCO_3_ and molybdenum-antimony antimetric colorimetry; total potassium was determined by NaOH melting and flame photometer; available potassium was determined by neutral ammonium acetate extraction and flame photometer.

### 2.3 Determination of soil enzyme activity

Soil enzymes were mainly determined as urease, sucrase, protease, acid phosphatase, cellulase, catalase, and polyphenol oxidase. Soil enzyme activities were determined using Enzyme Linked Immunosorbent Assay Kit (Shanghai Preferred Biotechnology Co., Ltd., Shanghai, China), and extracted and measured according to the instructions of different soil enzyme assay kits, with enzyme activities expressed in U/g. Briefly, 0.5 g of fresh soil was taken and extracted according to the steps in the kit instructions, and the absorbance of the extracted liquid was measured using a multifunctional enzyme labeling instrument (BioTek Synergy2 Gene 5, Vermont, USA) and then converted to enzyme activity. The absorbance was determined at 630 nm for urease, 540 nm for sucrase, 680 nm for protease, 660 nm for acid phosphatase, 540 nm for cellulase, 240 nm for catalase, and 430 nm for polyphenol oxidase. Three independent replicates were determined for each sample.

### 2.4 Soil macrogenome sequencing and bioinformatics analysis

Soil DNA was extracted using the Bio-Fast Soil Genomic DNA Extraction Kit (BioFlux, Hangzhou, China) according to the instruction manual of the kit, and the soil dosage was 0.5 g. The purity and integrity of DNA was examined by 1% agarose gel electrophoresis, and the concentration was determined by Qubit^®^ 2.0 Flurometer (Life Technologies, CA, USA) and then used for library construction.

The library was constructed by taking 1μg of DNA from each sample and randomly breaking it into fragments of about 350 bp using a Covaris Ultrasonic Crusher (E200, Woburn, MA, USA), and then generating sequencing libraries using NEB Next^®^Ultra™ DNA Library Prep Kit (NEB, Texas, USA) according to the instructions of the kit. After library construction, the products were purified using the AMPure XP system (Beckman Coulter, Beverly, USA). The purified products were initially quantified by Qubit 2.0, and then the libraries were diluted to 2 ng/μL, and then the insert size of the libraries was detected by Agilent 2100 (Agilent, California, USA). After the insertion size met the expectations, the effective concentration of the library was accurately quantified by real-time fluorescence quantitative PCR (the effective concentration of the library was > 3 nM) to ensure the quality of the library. The well-qualified libraries were entrusted to Wuhan Maiwei Metabolic Biotechnology Co., LTD (Wuhan, China) for sequencing using Illumina HiSep high-throughput sequencing platform with PE150 read length after pooling according to the effective concentration and target downstream data volume requirements.

Raw data obtained by sequencing were preprocessed using Fastp software (v0.20.1) to remove reads containing more than 40% of low-quality bases (quality value < 15), reads with more than 5 bp of N bases, adapter sequences, and sequences with a length of less than 15 bp to obtain clean data. Bowtie2 software (v2.3.4) was used to compare clean data with the host database and remove sequences that may have originated from the host, with software parameters set to -end-to-end, -sensitive, -I 200, -X 400 (Karlsson et al., 2012; Scher et al., 2013). The obtained clean data were assembled using MEGAHIT (v1.2.9) with parameters set to K-min 35, K-max 95, K-step 20, min-contig-len 500. The assembled contigs were compared using Bowtie2 (v2.3.4) with parameters set to -I 200, -X 400 to obtain unutilized PE reads, which were then subjected to gene prediction (Karlsson et al., 2012). Based on the contigs (≥500bp) obtained after assembly, the open reading frame (ORF) was predicted using MetaGeneMark (v3.38) (Ji et al., 2023). Based on the prediction results, predicted genes less than 100 nt in length were removed (Yones et al., 2021). The ORF prediction results were de-redundant using CD-HIT software (v4.8.1) to obtain a non-redundant initial gene catalogue (Kondratenko et al., 2020). Bowtie2 (v2.3.4) was used to compare the clean data of each sample with the initial unigenes, and the number of reads on which the gene was compared in each sample was calculated. The abundance of each gene in each sample was calculated based on the number of reads and gene length obtained from the comparison (Sun et al., 2020). Diamond software (v0.8.10) was used to align the obtained genes with sequences of bacteria, fungi, archaea, and viruses sampled from NCBI’s NR database (Blastp, evalue ≤ 1e-5) (Xu et al., 2023a). The annotated species were analyzed using the LCA algorithm of the MEGAN software to obtain the final species of the sequence (Portik et al., 2022). The abundance of a species is equal to the sum of the abundance of genes annotated to that species. The obtained gene sequences were aligned with the KEGG database using DIAMOND software (v 0.8.10) (Blastp, evalue ≤ 1e-5), and the alignment with the highest score was selected for subsequent analysis (Liu et al., 2020). The relative abundance of each functional tier is equal to the sum of the relative abundance of genes annotated to that functional tier.

### 2.5 Statistical analysis

Excel 2020 was used for preliminary statistical analysis of raw data and conventional bar graphs, and variance analysis (ANOVA) and paired Student ‘s t-tests were used to analyze differences in the different indexes, and differences were considered statistically significant when reaching the *p* < 0.05 level of significance. Rstudio software (v4.2.3) was used for in-depth analysis of the post-statistical data, including the production of graphs and the construction of models. The R package used for box plotting is gghalves 0.1.4, the R package used for vane diagrams is ggVennDiagram 1.5.2, the R package used for neutral community modeling is minpack.lm 1.2.4, the R package used for the α-diversity index and the β-diversity index is vegan 2.6.4, the R package used for modular network interaction diagrams is igraph 2.0.2, the R package of partial least squares structural squation modeling (PLS-SEM) equation is plspm 0.4.9, the R package of volcano maps is ggplot2 3.5.0, the R package of bubble feature maps is ggplot2 3.4.0, the R package used for technique for order preference by similarity to ideal solution (TOPSIS) is dplyr 1.1.4, the R package of orthogonal partial least squares discrimination analysis (OPLS-DA) model construction is ropls and mixOmics, the R package of KEGG function enrichment is clusterProfiler 4.10.0, the R package of redundancy analysis is vegan 2.6.4, and the R package of interaction network map is linkET 0.0.7.1 (Wickham, 2019).

## 3 Results and discussion

### 3.1 Analysis of basic physicochemical indexes and soil enzyme activities of rhizosphere soils of tea tree

**Figure 1:**
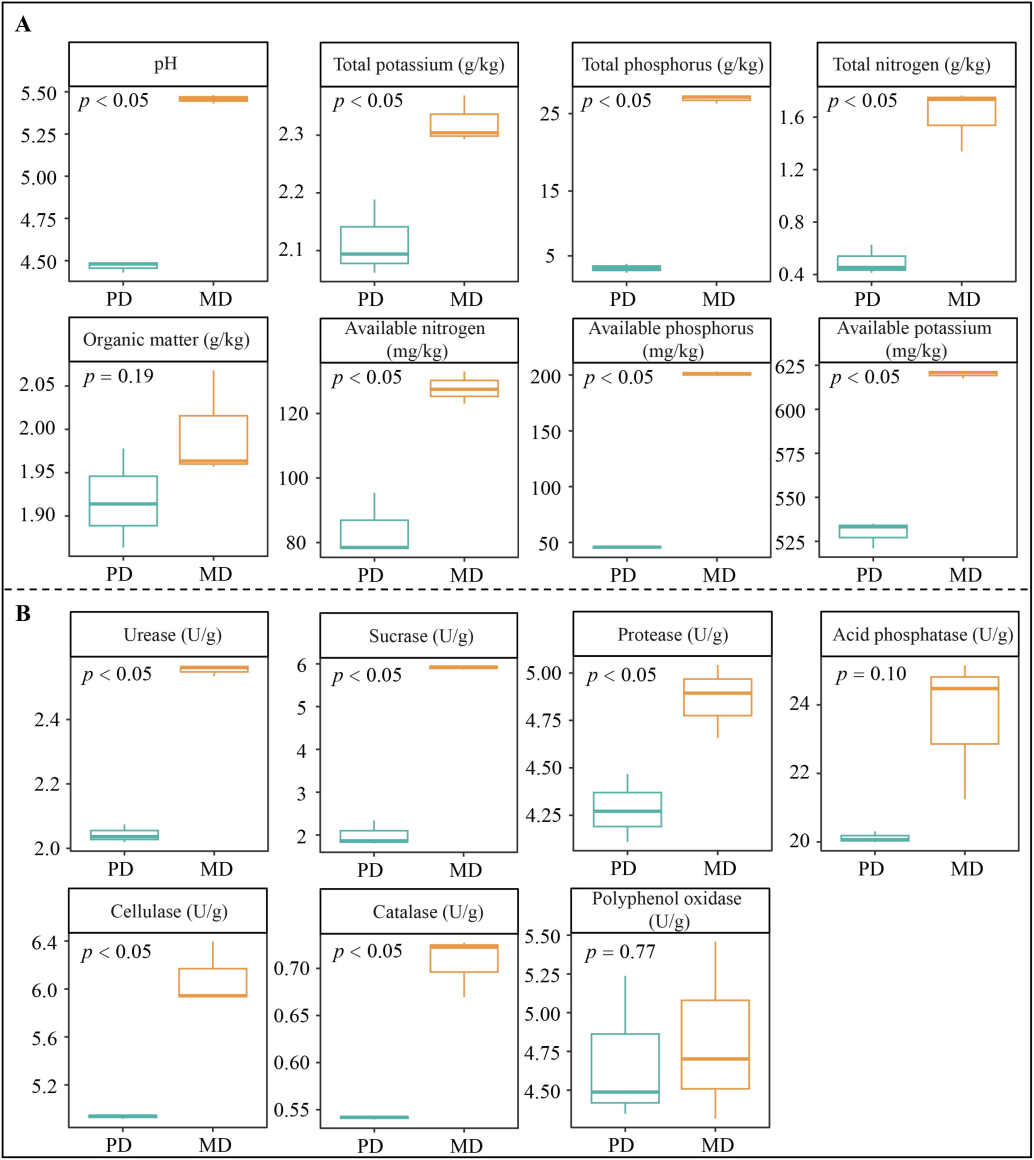
Basic physicochemical indexes and soil enzyme activities in rhizosphere soil of tea trees. MD: Dahongpao mother tree; PD: Cutting Dahongpao; **(A)** Soil basic physicochemical indexes; **(B)** Soil enzyme activities.

The tea tree is an acidophilic plant. When soil pH is 4.5 to 5.5, it is suitable for tea tree growth, and when pH is 5.0 to 5.5, it is most suitable for tea tree growth (Yang et al., 2024). Changes in soil pH affect enzyme activity in the soil, which in turn affects the ability to transform soil nutrients and alters soil nutrient content (Borase et al., 2020). Nutrient supply is required for plant growth, and high or low soil nutrient content, especially available nutrient content, directly affects nutrient uptake and hence plant growth (Soares et al., 2019). In this study, rhizosphere soil pH values of Dahongpao mother tree (MD) and cutting Dahongpao (PD) were 5.46 and 4.47, respectively (**Figure 1A**). The contents of total nitrogen, total phosphorus, total potassium, available nitrogen, available phosphorus and available potassium were significantly higher in rhizosphere soil of MD than in PD by 3.24, 5.38, 1.10, 1.52, 4.42 and 1.17 times, respectively. As for organic matter content, the difference between MD and PD was not significant. Analysis of soil enzyme activity showed (**Figure 1B**) that all rhizosphere soil enzyme activities were higher in MD than in PD, with urease, sucrase, protease, cellulase, and catalase activities being significantly higher in MD than in PD, as evidenced by 1.25-, 2.95-, 1.14-, 1.23-, and 1.30-fold higher, respectively. Soil enzyme activities can influence soil nutrient conversion capacity, with higher activities favoring higher available nutrient content of the soil (Zielewicz et al., 2021). Soil pH can change the enzyme activity in the soil, and with the increase of soil pH, the enzyme activity of the rhizosphere soil of tea tree rises significantly, the nutrient conversion capacity of the soil is enhanced, the content of available nutrients rises, and the yield and quality of the tea tree are significantly higher (Jia et al., 2023). It can be seen that rhizosphere soil pH of MD was more favorable for tea tree growth compared to PD; secondly, the content of total N, P, K and available N, P and K in rhizosphere soil of MD was significantly higher than that of PD. Therefore, under more suitable soil pH conditions, the enzyme activity of MD rhizosphere soil is significantly enhanced, which is more conducive to soil nutrient transformation and more conducive to increasing the available nutrient content of the soil, which in turn promotes tea tree uptake and facilitates tea tree growth.

### 3.2 Macrogenome sequencing results of rhizosphere soil microorganisms of tea tree

**Figure 2:**
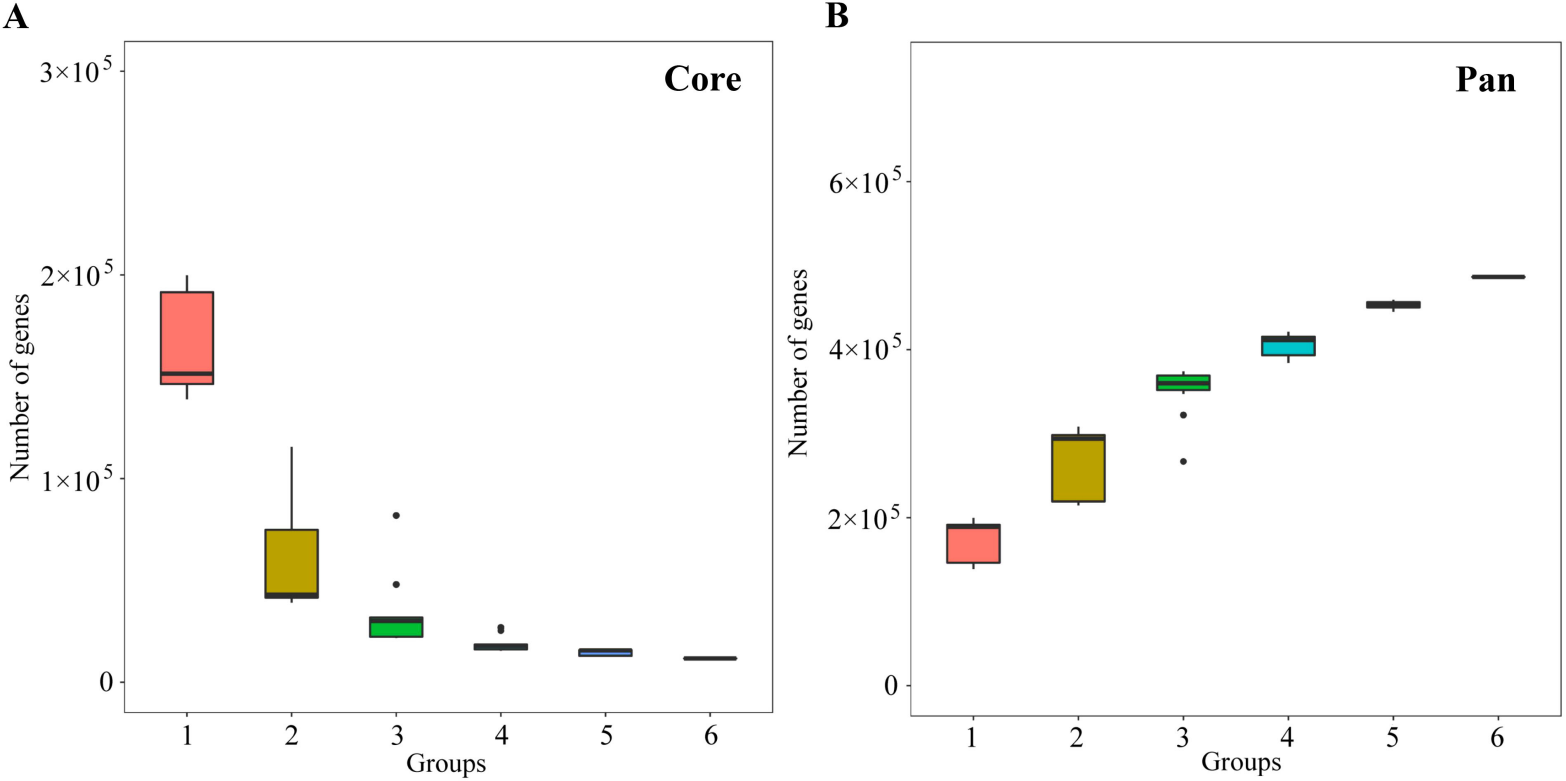
Core and pan gene dilution curve analysis of soil samples. **(A)** Dilution curve of core gene; **(B)** Dilution curve of pan gene.

Rhizosphere soil macro-genomes of MD and PD were sequenced and analyzed using Illumina, and a total of 42.64 G of clean data was obtained for the six samples (**Supplementary Table S1**). MEGAHIT (v1.2.9) software was used to assemble clean data, and a total of 821,690 contigs were obtained, with a total length of 617,325,279 bp (**Supplementary Table S2**). MetaGeneMark (v3.38) software was used for open reading frame prediction of contigs, and a total of 262,247 genes were obtained, and the total length of genes in the gene catalogue was 386.75 Mbp, with an average length of 417.47 bp (**Supplementary Table S3**). Further analysis of core and pan gene dilution curves for the obtained genes revealed that core genes decreased with increasing number of samples and stabilized when the number of samples reached 4 (**Figure 2A**), while pan genes increased with increasing number of samples and stabilized when the number of samples reached 5 (**Figure 2B**). Core and pan gene dilution curves can be used to assess gene stability in samples, in which the core and pan gene curves tend to be stable, indicating that the number of genes is gradually stabilized, the number of new genes is small, and the sample test results meet the requirements to cover a large range (Xu et al., 2023b). It can be seen that the soil macro-genome sequencing results of this study can basically reflect the microbial communities in the soil, and the sequencing results can be used for further in-depth analysis.

### 3.3 Analysis of microbial diversity in rhizosphere soil of tea tree

**Figure 3:**
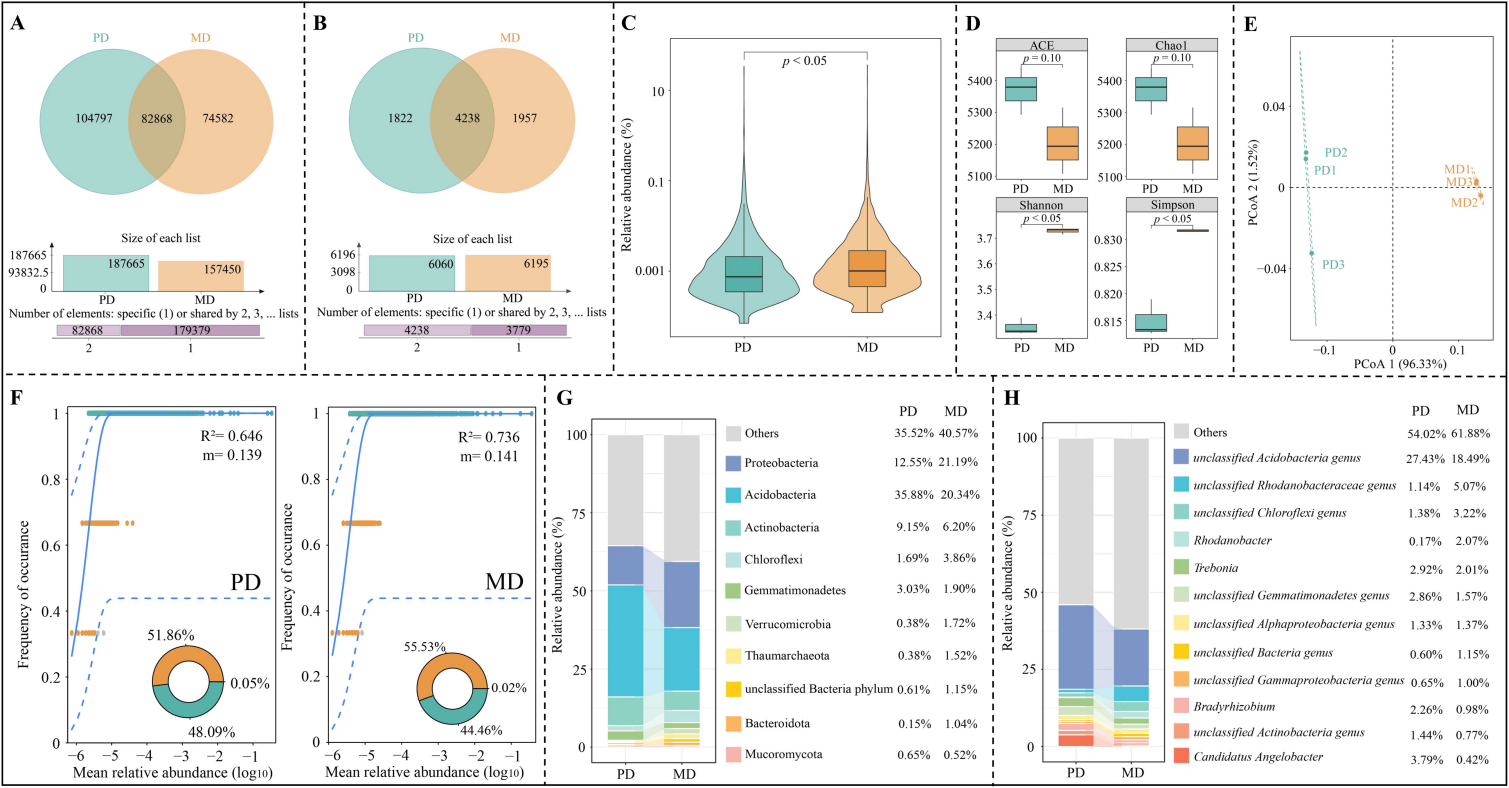
Microbial diversity analysis of rhizosphere soil of tea tree. MD: Dahongpao mother tree; PD: Cutting Dahongpao; **(A)** Vane diagram analysis of soil genes; **(B)** Vane diagram analysis of soil microorganisms; **(C)** Analysis of variance of total abundance of soil microorganisms; **(D)** Analysis of α-diversity index; **(E)** PCoA analysis of β-diversity index; **(F)** Analysis of neutral community model of soil microorganisms; **(G)** Top ten soil microorganisms in terms of abundance at the phylum level; **(H)** Top ten soil microorganisms in terms of abundance at the genus level.

Macro-genome sequencing of tea tree rhizosphere soil (**Figure 3A**) showed that a total of 262,247 genes were detected in rhizosphere soil, of which 82,868 genes were shared by PD and MD, 104,797 genes were specific to PD, and 74,582 genes were specific to MD. Species annotation based on the obtained genes showed (**Figure 3B**) that a total of 8,018 microbial species were annotated, of which 4,329 species were shared by PD and MD, 1,822 species were unique to PD, and 1,957 species were unique to MD. Microbial species abundance analysis showed (**Figure 3C**) that there was a significant difference in microbial species abundance between PD and MD. It is evident that there were some differences in the number and abundance of genes and microorganisms in the rhizosphere soils of MD and PD, which in turn may affect the diversity and abundance of their microbial species.

Microbial diversity is generally assessed using the α-diversity and β-diversity indexes. In α-diversity indexes, ACE and Chao1 indexes can be used to assess species richness and evenness, and Simpson and Shannon indexes can be used to assess the diversity of species within a sample (Willis, 2019). Species diversity differences between samples were assessed using the β-diversity index (Xiao et al., 2021). Therefore, the microbial community of rhizosphere soil of tea tree was further analyzed in this study using α-diversity and β-diversity indexes. Analysis of the α-diversity indexes showed (**Figure 3D**) that both Shannon and Simpson indexes were significantly higher for MD than for PD (*p* < 0.05), whereas for the ACE and Chao1 indexes, there was no significant difference between MD and PD (*p* > 0.05). PCoA analysis of the β-diversity index showed (**Figure 3E**) that the two principal components could effectively differentiate between MD and PD with an overall contribution of 97.85%. It can be seen that rhizosphere soil microorganisms in MD and PD differed less in abundance and evenness, whereas there were significant differences in diversity, which in turn may alter their community structure.

Neutral community model can be used to assess the stability, complexity, and spread degree of microbial community among different samples (Yang et al., 2022). In this study, neutral community models of rhizosphere soil microorganisms in MD and PD found (**Figure 3F**) that MD had higher R^2^ (0.736) and migration rate (m, 0.141) values than PD (R^2^ = 0.646, m = 0.139). It can be seen that the MD rhizosphere soil microbial community has a high spread degree and a more complex community structure. Accordingly, this study further analyzed the microbial community structure in MD and PD and found that at the phylum level (**Figure 3G**), the top three phyla of microorganisms with the largest percentage in rhizosphere soils of MD and PD were Acidobacteria, Proteobacteria, and Actinobacteria, with 20.34%, 21.19%, and 6.20% in MD, 35.88%, 12.55%, and 9.15% in PD, respectively. At the genus level (**Figure 3H**), the top three genera of microorganisms with the highest percentage in MD rhizosphere soil were *unclassified Acidobacteria genus* (18.49%), *unclassified Rhodanobacteraceae genus* (5.07%), *unclassified Chloroflexi genus* (3.22%), while the top three genera of microorganisms in PD rhizosphere soil was *unclassified Acidobacteria genus* (27.43%), *Candidatus Angelobacter* (3.79%), *Trebonia* (2.92%). It can be seen that the richness and diversity of soil microorganisms in the rhizosphere of MD were higher than those of PD, and its rhizosphere soil microbial community had a higher spread degree and a more complex community structure.

### 3.4 Nodal analysis of microbial modular interaction networks

**Figure 4:**
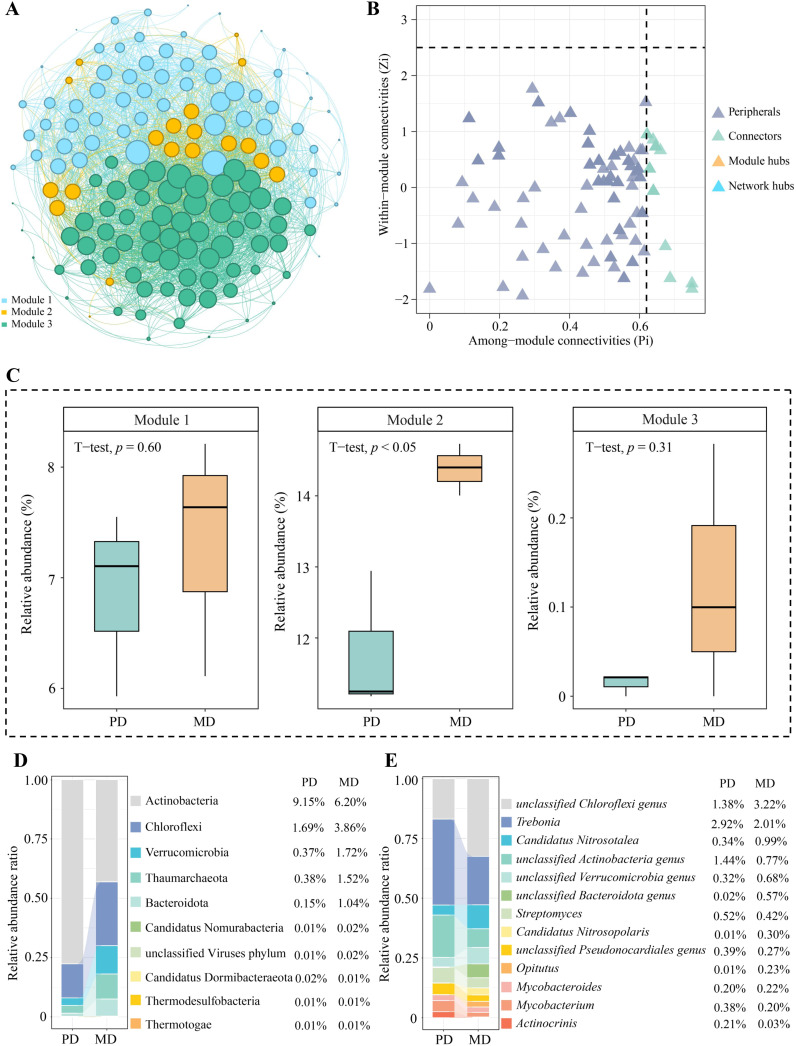
Modular interaction network node analysis of soil microorganisms in rhizosphere of tea tree. MD: Dahongpao mother tree; PD: Cutting Dahongpao; **(A)** analysis of modular interaction network of microorganisms; **(B)** analysis of keystone nodes of modular networks; **(C)** analysis of species abundance of keystone nodes of different modules; **(D)** The top ten microorganisms of keystone nodes in terms of abundance at phylum level; **(E)** The top ten microorganisms of keystone nodes in terms of abundance at genera level.

**Figure 5:**
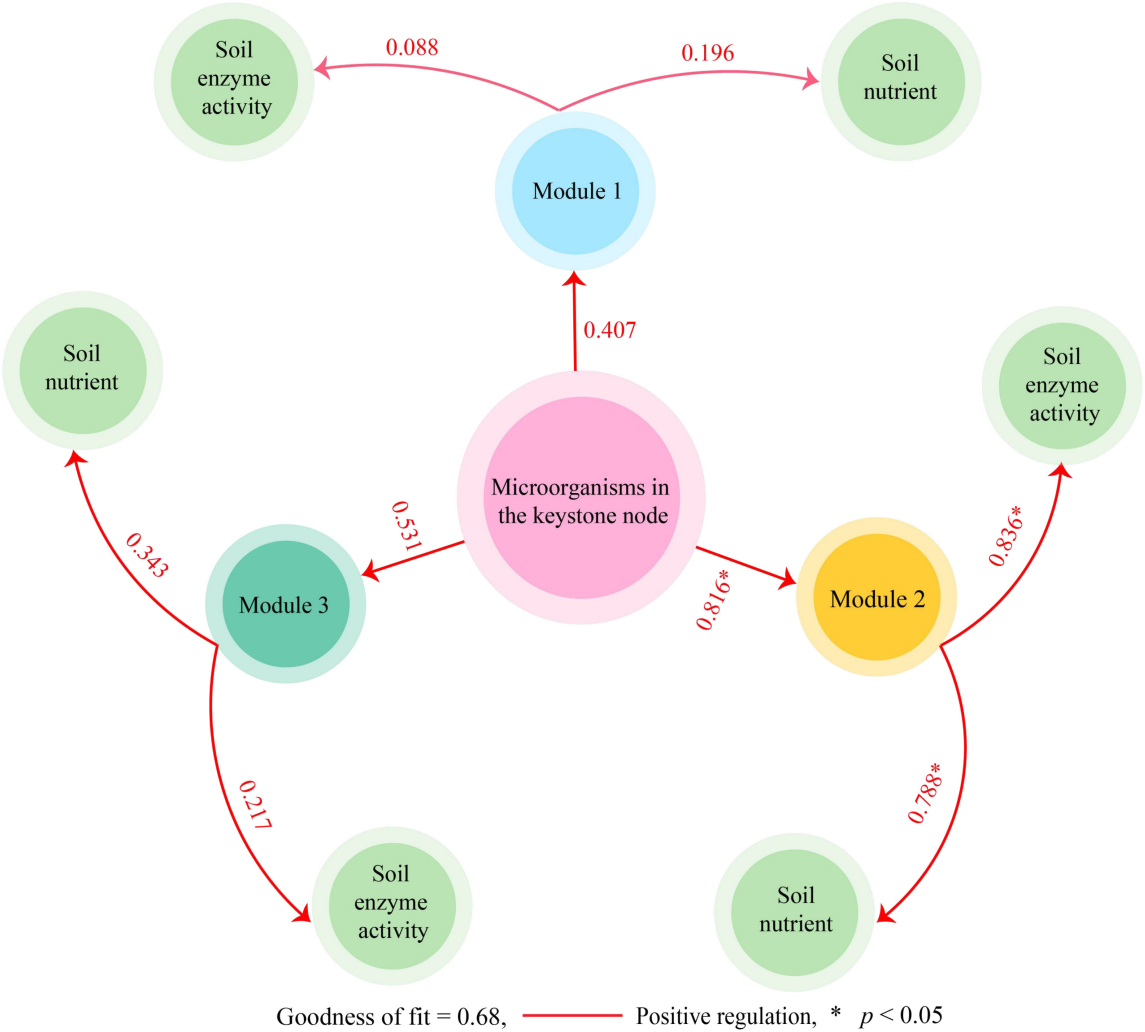
PLS-SEM equations of of microorganisms of different modules with soil basal physicochemical indexes and enzyme activities. “*” indicates a significant correlation between the two variables.

Direct or indirect interactions between coexisting microorganisms in microbial community structure analysis can indicate functional roles or environmental ecological niches for microorganisms in the environment (Siles et al., 2021). Network analysis of microorganisms using modularization to create taxonomic modules of coexisting microbial taxa and finding keystone nodes with high connectivity in the modular network can lead to microbial communities with direct relationships (Xun et al., 2021). Based on the above analysis, the identified microbial species were categorized in this study, mainly from 146 phyla. Using modular interaction network analysis, it was found (**Figure 4A**) that 146 phyla could be distinguished into three modules, with modules 1, 2, and 3 containing 62, 19, and 65 phyla, respectively. Further topological role analysis of the nodes in the three modular networks revealed (**Figure 4B**) that there were a total of 20 keystone nodes in the three modules and all of them were highly connected nodes (Connector, Zi < 2.5, Pi > 0.6), with 8, 11, and 1 phyla in module 1, 2, and 3, respectively. Microbial abundance analysis of keystone nodes in different modules showed (**Figure 4C**) that PD was not significantly different from MD for microbial abundance in module 1 and module 3, while for the microbial abundance of module 2, MD was significantly greater than PD. Therefore, this study further analyzed the microbial community structure of the 11 keystone nodes in module 2 and showed that at the phylum level (**Figure 4D**), the top three phyla with the largest percentage of microorganisms in the rhizosphere soil of MD were Acidobacteria (6.20%), Chloroflexi (3.86%), and Verrucomicrobia (1.72%), while in PD they were Acidobacteria (9.15%), Chloroflexi (1.69%), Thaumarchaeota (0.38%). At the genus level (**Figure 4E**), the top three genera of microorganisms with the largest percentage in the MD rhizosphere soil were *unclassified Chloroflexi genus* (3.22%), *Trebonia* (2.01%), *Candidatus Nitrosotalea* (0.99%), and in PD, they were *Trebonia* (2.92%), *unclassified Actinobacteria genus* (1.44%), *unclassified Chloroflexi genus* (1.38%). In addition, this study further constructed PLS-SEM equations of microorganisms of the three modules with soil basal physicochemical indexes and enzyme activities, and the results showed (**Figure 5**) that microorganisms of all 20 keystone nodes could positively regulate the three modules, whereas only module 2 was regulated at a significant level (0.816*). Secondly, all three modules positively regulated soil nutrients and enzyme activities, while only module 2 regulated soil nutrients (0.788*) and enzyme activities (0.836*) at a significant level. It can be seen that the microorganisms of the keystone node in module 2 can significantly affect soil nutrient transformation and enzyme activities, and are the key microorganisms that distinguish MD from PD.

### 3.5 Screening for characteristic microorganisms

**Figure 6:**
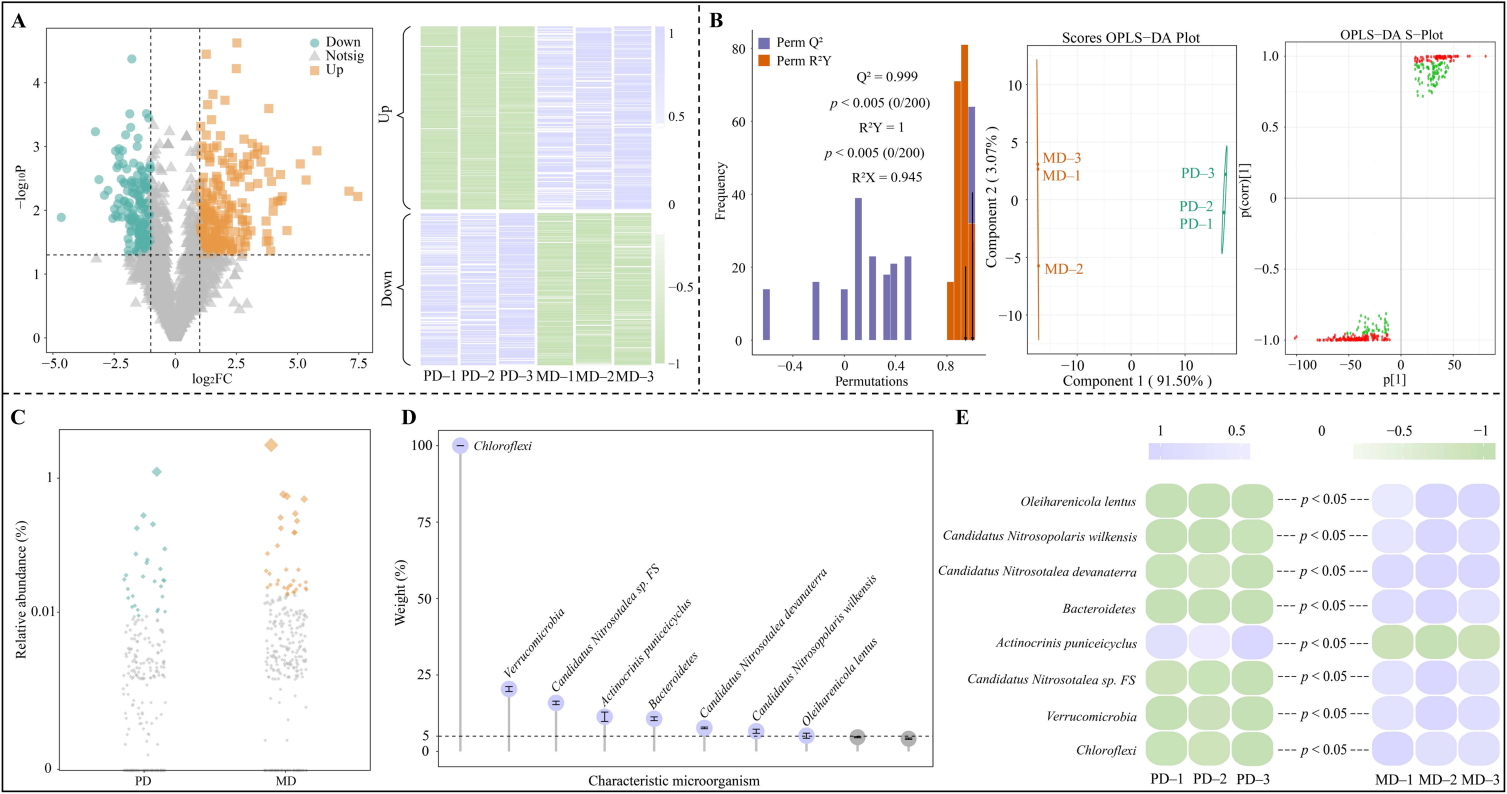
Screening of characteristic microorganisms in the rhizosphere soil of tea tree. MD: Dahongpao mother tree; PD: Cutting Dahongpao; **(A)** Volcano map to screen microorganisms significantly different from MD and PD and their abundance; **(B)** OPLS-DA model construction of MD and PD to screen key different microorganisms; **(C)** Bubble feature map to screen characteristic microorganisms; **(D)** the contribution of characteristic microorganisms to differentiating MD and PD by TOPSIS analysis; **(E)** Abundance analysis of characteristic microorganisms.

Based on the above analysis, this study was conducted with microorganisms from 11 keystone nodes in module 2 to screen for characteristic microorganisms with significant differences. The 11 keystone nodes in module 2 involved a total of 2804 microbial species, which were analyzed using volcano diagram and found (**Figure 6A**) that 392 microbial species were significantly different in MD and PD, of which the abundance of 214 microbial species was significantly greater in MD than in PD, and that of 178 microbial species was significantly less in MD than in PD. The OPLS-DA model of MD and PD was constructed to analyze 392 microorganisms with significant differences to screen for key microorganisms, and the results showed (**Figure 6B**) that the fit and predictability of the constructed model reached a significant level (R^2^Y = 1, *p* < 0.005; Q^2^ = 0.999, *p* < 0.005), and it was effective in distinguishing MD from PD. The S-Plot plots of the OPLS-DA model screened for 258 key microorganisms with importance projection values (VIP) greater than 1. Further analysis of 258 microorganisms using bubble feature maps revealed (**Figure 6C**) that there were 50 characteristic microorganisms with significant differences between MD and PD. Accordingly, in this study, TOPSIS was used to analyze the characteristic microorganisms with significant differences to determine the contribution of different microorganisms in distinguishing MD from PD, and the results showed (**Figure 6D**) that only eight microorganisms had a contribution of more than 5%, namely, *Chloroflexi*, *Verrucomicrobia*, *Candidatus Nitrosotalea* sp *FS*, *Actinocrinis puniceicyclus*, *Bacteroidetes*, *Candidatus Nitrosotalea devanaterra*, *Candidatus Nitrosopolaris wilkensis* and *Oleiharenicola lentus*. Abundance analysis of the eight characteristic microorganisms showed (**Figure 6E**) that only the abundance of *Actinocrinis puniceicyclus* was significantly less in MD than in PD, and for the remaining seven characteristic microorganisms, MD was significantly greater than PD.

*Verrucomicrobia* (Bünger et al., 2020) and *Oleiharenicola lentus* (Chung et al., 2022) have been reported to play a key role in the biodegradation of hazardous substances in soils, which can ameliorate the soil for plant growth. *Chloroflexi* is associated with carbon and nitrogen cycling in the soil and can improve soil carbon and nitrogen utilization (Xue et al., 2020). *Candidatus Nitrosotalea devanaterra* (Bei et al., 2024) and *Candidatus Nitrosopolaris wilkensis* (Pessi et al., 2022) play an important role in the oxidation of ammonia in the soil, which enhances the nitrogen cycling capacity of the soil and consequently converts nitrogen from organic to soluble form. *Bacteroidetes* has the ability to secrete phosphatase, which is an important contributor to soil phosphorus activation and is effective in enhancing soil phosphorus cycling and increasing soil available phosphorus content (Lidbury et al., 2021). In this study, the abundance of *Verrucomicrobia* and *Oleiharenicola lentus* was found to be significantly greater in MD than in PD, and it is evident that rhizosphere soil microorganisms of MD were more capable than those of PD in degrading soil hazardous substances and ameliorating soil. Second, under favorable environmental conditions, the abundance of *Chloroflexi*, *Candidatus Nitrosotalea*, *Candidatus Nitrosotalea devanaterra*, *Candidatus Nitrosopolaris wilkensis* and *Bacteroidetes* in soils was significantly higher in MD than in PD. It can be seen that the rhizosphere soil of MD had a higher biotransformation capacity for carbon, nitrogen and phosphorus compared to PD, which was more conducive to the accumulation and release of nutrients in the soil, and more conducive to the promotion of tea tree growth. In addition, this study also found that the abundance of *Actinocrinis puniceicyclus* was significantly lower in rhizosphere soil of MD compared to PD. *Actinocrinis puniceicyclus* is an acidophilic actinomycete with antibiotic-producing ability, but the higher the environmental pH, the weaker its ability to colonize (Kusuma et al., 2022). Rhizosphere soil pH was also higher in MD in this study, which again may have led to reduced colonization and lower abundance of *Actinocrinis puniceicyclus*.

### 3.6 Functional analysis of characteristic microorganisms

**Figure 7:**
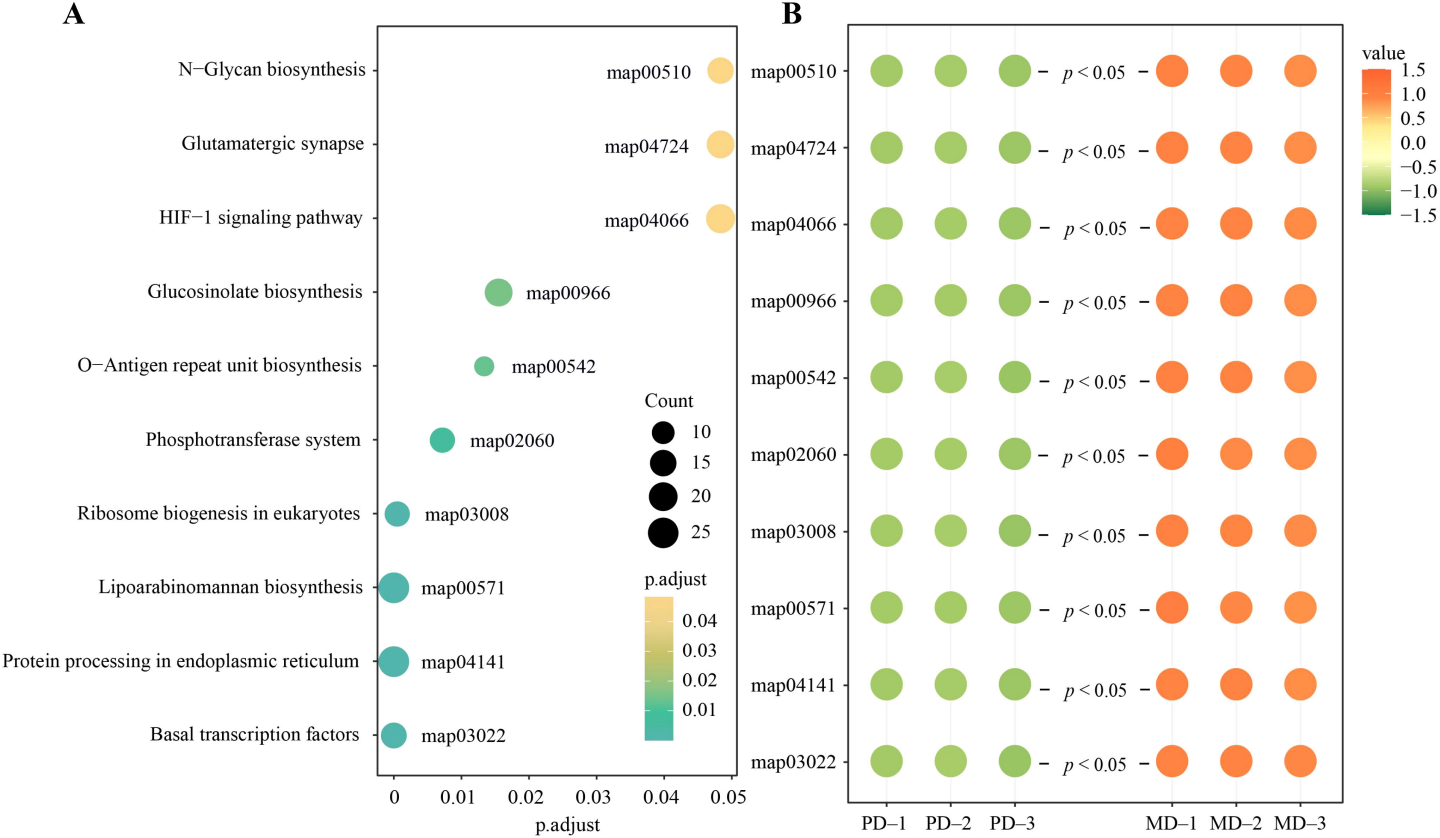
Functional analysis of characteristic microorganisms. **(A)** Enrichment analysis of characteristic microbial functions; **(B)** Microbial abundance analysis of functionally significant enrichment pathways.

Based on the above analysis, functional enrichment analysis was performed with the eight characteristic microorganisms, and the results showed (**Figure 7A**) that 10,661 genes corresponding to the eight characteristic microorganisms were enriched by the annotated gene functions, and a total of 163 pathways were enriched, of which 10 were significantly enriched, namely, N-glycan biosynthesis (map00510), glutamatergic synapse (map04724), HIF-1 signaling pathway (map04066), glucosinolate biosynthesis (map00966), O-antigen repeat unit biosynthesis (map00542), phosphotransferase system (map02060), ribosome biogenesis in eukaryotes (map03008), lipoarabinomannan biosynthesis (map00571), protein processing in endoplasmic reticulum (map04141), basal transcription factors (map03022). Further analysis showed (**Figure 7B**) that the abundance of 10 significantly enriched pathways was all significantly greater for MD than for PD.

Enhancement of the HIF-1 signaling pathway has been reported to induce a range of physiological adaptive responses in microorganisms to adapt to their environment (Ba et al., 2022). Enhancement of basal transcription factors and ribosome biogenesis in eukaryotes accelerates microbial transcription and translation and increases the rate of microbial reproduction. Products of O-Antigen repeat unit biosynthesis promote cell proliferation (Mikkola, 2020), protein processing in endoplasmic reticulum promotes cell synthesis (Wiseman et al., 2022), and products of N- glycan biosynthesis promote cell differentiation (Tolonen et al., 2022). Products of Glucosinolate biosynthesis have the effect of inducing and aggregating microorganisms to improve soil microbial community structure (Chroston et al., 2024). It can be seen that the microorganisms in rhizosphere soil of MD are more environmentally adapted, with faster reproduction rates and greater aggregation capacity than in PD, which in turn contributes to higher diversity and abundance of microorganisms. Secondly, this study also revealed that the abundance of glutamatergic synapse, lipoarabinomannan biosynthesis and phosphotransferase system pathways was significantly higher in MD rhizosphere soil than in PD. Products of Lipoarabinomannan biosynthesis resist soil pathogens and enhance soil carbon and nitrogen metabolism (Wang et al., 2023b). Glutamatergic synapse promotes soil nitrogen cycling and improves soil nutrient biotransformation (Jiang et al., 2020). Whereas the enhanced function of the phosphotransferase system accelerates the mineralization of organic phosphorus in the soil, thus directly increasing the effectiveness of phosphorus in the soil (Wang et al., 2024b). It can be seen that the rhizosphere microorganisms of MD were significantly higher than those of PD in the biotransformation of soil carbon, nitrogen, and phosphorus, and were more favorable for nutrient transformation and accumulation in the soil.

### 3.7 Interaction network analysis between characteristic microorganisms and different indexes

**Figure 8:**
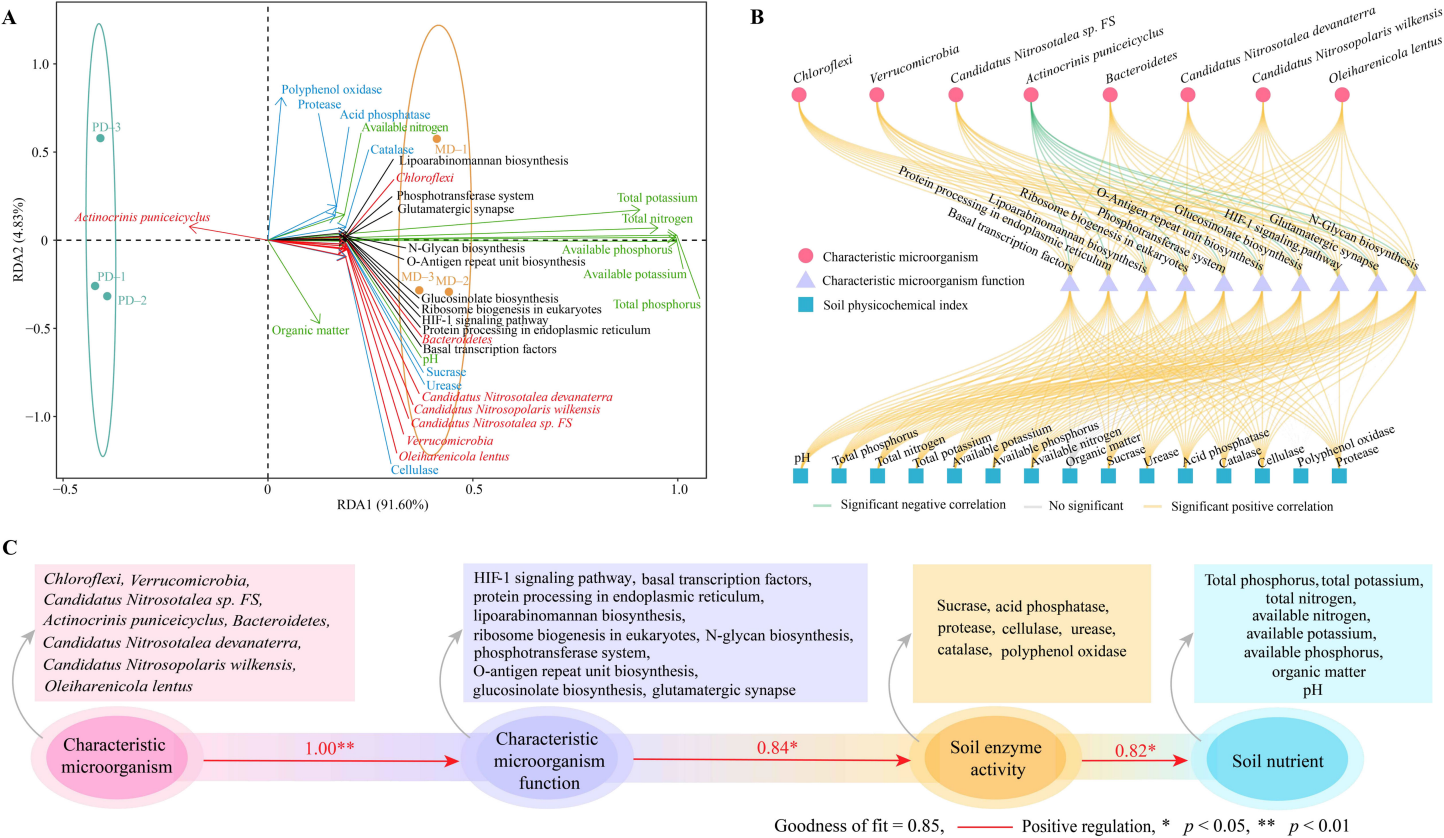
Interaction analysis between different indexes. MD: Dahongpao mother tree; PD: Cutting Dahongpao; **(A)** RDA analysis of characteristic microorganisms and functions with different indexes; **(B)** Correlation network analysis of characteristic microorganisms and functions with different indexes; **(C)** Construction of PLS-SEM equations for characteristic microorganisms, microbial functions, soil enzyme activities and soil nutrients.

Based on the above analysis, this study further performed the interaction analysis of characteristic microorganisms and their functions with different indexes. RDA analysis showed (**Figure 8A**) that 8 characteristic microorganisms, except *Actinocrinis puniceicyclus*, the remaining 7 characteristic microorganisms were correlated with the characteristic microbial functions of rhizosphere soil, and soil physicochemical indexes. Whereas the 7 characteristic microorganisms mentioned above were associated with MD, only *Actinocrinis puniceicyclus* was associated with PD. Correlation network analysis showed (**Figure 8B**) that characteristic microorganisms such as *Chloroflexi*, *Verrucomicrobia*, *Candidatus Nitrosotalea* sp *FS*, *Bacteroidetes*, *Candidatus Nitrosotalea devanaterra*, *Candidatus Nitrosopolaris wilkensis* and *Oleiharenicola lentus* were significantly positively correlated with soil microbial functions, while *Actinocrinis puniceicyclus* was significantly negatively correlated. Secondly, soil microbial functions were significantly and positively correlated with soil physicochemical indexes, except for organic matter content where the correlation did not reach a significant level. The PLS-SEM equations between characteristic microorganisms, microbial functions, soil enzyme activities and soil nutrients were further constructed, and the results showed (**Figure 8C**) that characteristic microorganisms positively regulated soil microbial functions (1.00**), positively regulated soil enzyme activities (0.84*), and positively regulated soil nutrient content (0.82*). It can be seen that the abundance of soil characteristic microorganisms in rhizosphere soil of MD increased significantly compared with that of PD, prompting a significant enhancement of their corresponding functions, which was more conducive to soil improvement, increased soil enzyme activity, enhanced the biotransformation of soil nutrients, and then increased the accumulation and effectiveness of soil nutrients to promote the growth of tea trees.

## 4 Conclusion

**Figure 9:**
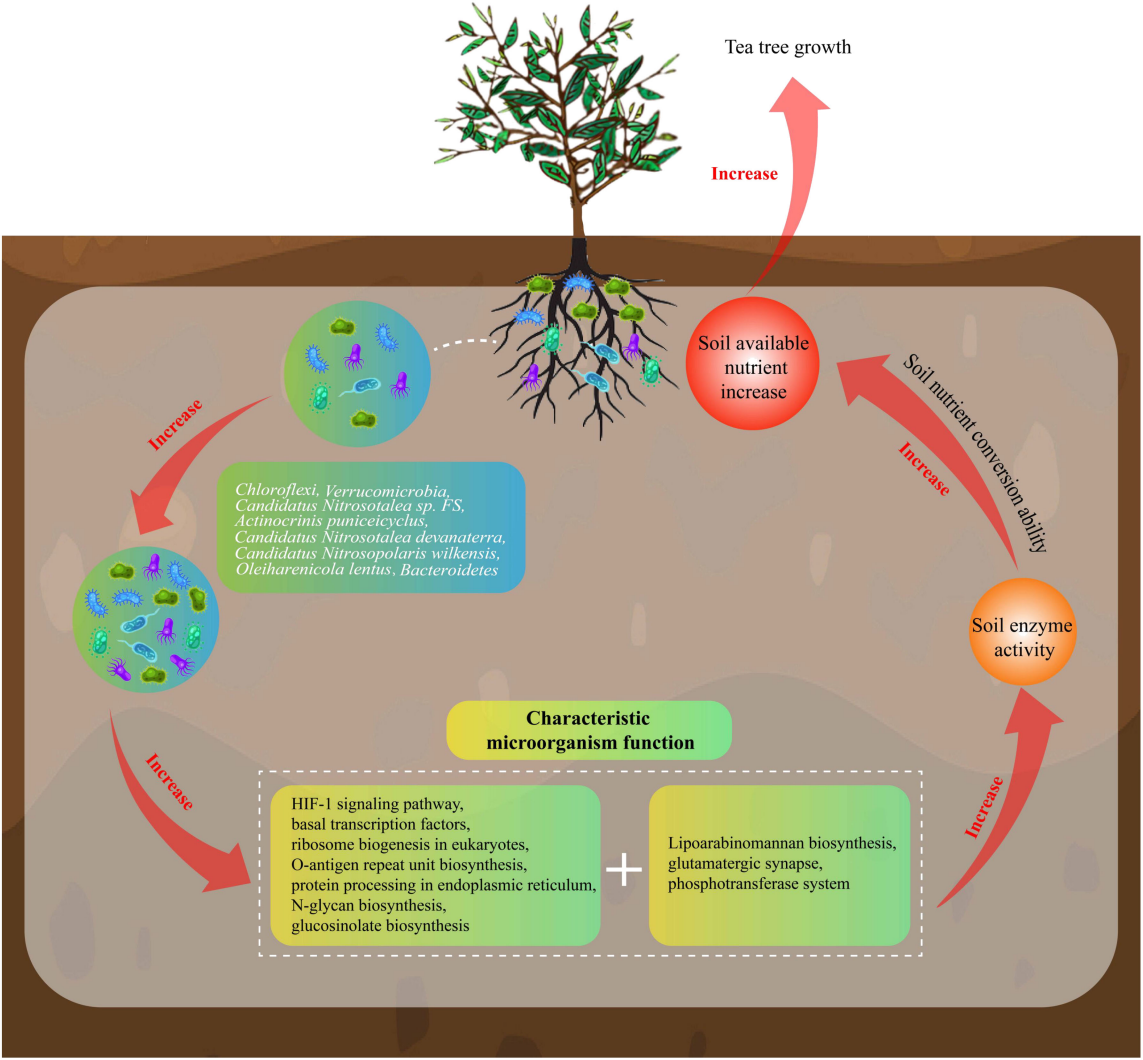
Effects of soil characteristic microorganisms and their functions on nutrient cycling in rhizosphere soil of tea tree.

In this study, we analyzed the effect of microbial diversity and its function on rhizosphere soil nutrients of MD and PD and found (**Figure 9**) that soil pH of MD was more favorable for tea tree growth compared to PD. Under more suitable soil pH conditions, the enzyme activity of MD rhizosphere soil was significantly enhanced, which was more conducive to the transformation of soil nutrients, more conducive to increasing the available nutrient content of the soil, which in turn promoted tea tree uptake and facilitated tea tree growth. Secondly, rhizosphere soil microbial richness and diversity were higher in MD than in PD, and rhizosphere soil microbial community had a higher degree of spread and a more complex community structure. There were 8 characteristic microorganisms that differed significantly between MD and PD rhizosphere soils, and functional analysis showed that the microorganisms in MD rhizosphere soils were more environmentally adapted, with faster reproduction rates and greater aggregation capacity, which in turn contributed to higher diversity and abundance of microorganisms. MD rhizosphere soil had a higher capacity for carbon, nitrogen and phosphorus biotransformation, which was more conducive to the accumulation and release of nutrients in the soil, and more conducive to promoting tea tree growth. PLS-SEM equation analysis showed that the characteristic microorganisms positively regulated soil microbial functions, positively regulated soil enzyme activities, and positively regulated the nutrient content of the soil. In conclusion, the abundance of soil characteristic microorganisms in rhizosphere soil of MD increased significantly compared with PD, which led to a significant enhancement of their corresponding functions, and was more conducive to the improvement of the soil, such as increasing the activity of soil enzymes, enhancing the biotransformation capacity of soil nutrients, and increasing the accumulation and effectiveness of soil nutrients, thus promoting tea tree growth. Therefore, in the process of planting management, the soil acidity and alkalinity of tea tree cuttings should be adjusted to avoid their soil pH being too low, and at the same time, the soil should be loosened and fertilized at the right time to improve the soil permeability, so as to enhance the diversity of soil microorganisms and nutrient conversion capacity, which is conducive to the growth of the tea tree to guarantee tea yield and quality.

## Data Availability

Data will be made available on request. The original contributions of Metagenomic data presented in the study were publicly available. This date can be found here: NCBI, PRJNA1155230421 (https://www.ncbi.nlm.nih.gov/bioproject/?term=PRJNA1155230).
