# Supplementary material for: Effect of microbial diversity and their functions on soil nutrient cycling in the rhizosphere zone of Dahongpao mother tree and cutting Dahongpao
Source: Front Plant Sci. 2025 May 8;16:1574020. doi: 10.3389/fpls.2025.1574020 (PMC12095365; doi:10.3389/fpls.2025.1574020)
Supplement: Supplementary file 1 [file DataSheet1.pdf]

Supplementary Materials

Table S1 Basic information about sequencing data

Sample	CleanData bases (G)	CleanData Q20	CleanData Q30	CleanData GC
PD–1	7.32	98.14	94.81	61.18
PD–2	7.02	98.03	94.53	61.07
PD–3	7.23	98.08	94.67	61.46
MD–1	6.89	98.09	94.69	61.2
MD–2	6.76	98.08	94.65	61.27
MD–3	7.42	98.16	94.84	61.2

Note: MD: Dahongpao mother tree; PD: Cutting Dahongpao; Sample: Sample name; CleanData: Indicates valid data obtained by filtering; Bases: Number of bases in the data; Q20: Represents the percentage of the number of bases in the data with a sequencing error rate of less than 0.01(mass value greater than 20); Q30: Represents the percentage of the number of bases in the data with a sequencing error rate less than 0.001(mass value greater than 30); GC: GC content of bases in the data.

Table S2 The basic information of contigs after assembly of each

Assembly	Sample					
	PD–1	PD–2	PD–3	MD–1	MD–2	MD–3
Contigs (≥ 0 bp)	171891	157816	164142	110020	100869	116952
Contigs (≥ 500 bp)	171891	157816	164142	110020	100869	116952
Contigs (≥ 1000 bp)	23709	21654	21051	10618	9306	11058
Contigs (≥ 5000 bp)	383	352	306	122	128	114
Contigs (≥ 10000 bp)	79	83	66	15	23	17
Contigs (≥ 25000 bp)	2	5	4	0	0	1
Contigs (≥ 50000 bp)	1	0	0	0	0	0
Length (≥ 0 bp)	134186608	123112753	125377852	78865607	72089106	83693353
Length (≥ 500 bp)	134186608	123112753	125377852	78865607	72089106	83693353
Length (≥ 1000 bp)	39299742	36048260	33916682	16191344	14471061	16924812
Length (≥ 5000 bp)	3188688	3003422	2589586	895937	1017599	902587
Length (≥ 10000 bp)	1239378	1255583	1004018	199712	318044	277263
Length (≥ 25000 bp)	98906	160245	145037	0	0	31969
Length (≥ 50000 bp)	69026	0	0	0	0	0
Total contigs	171891	157816	164142	110020	100869	116952
Largest contig	69026	34974	46831	19639	23437	31969
Total length	134186608	123112753	125377852	78865607	72089106	83693353
GC (%)	60.49	60.32	60.97	60.26	60.24	60.52
N50	725	724	711	667	663	665
N75	581	580	576	562	561	563
L50	57015	52311	56022	40239	36926	42891
L75	109273	100325	105454	72672	66698	77337

Note：MD: Dahongpao mother tree; PD: Cutting Dahongpao; Sample: Sample name; Contigs: Indicates the number of contigs obtained by assembly; Length: Represents the length of the assembled contigs; Total contigs: The total number of contigs assembled; Largest contig: Indicates the maximum length of contigs; Total length: Indicates the total length of contigs assembled; N50(N75): Indicates that contigs are sorted by length, and then the sum is added from length to length, when the sum value reaches 50%(75%) of the total length of contigs; L50(L75): Indicates the number of contig when it reaches N50(N75).

Table S3 Basic information about Unigenes

List	Unigenes
ORFs number	262247
Integrity-start	85972 (32.78%)
Integrity-end	78498 (29.93%)
Integrity-none	29533 (11.26%)
Integrity-all	68244 (26.02%)
Total Length (Mbp)	386.75
Average Length (bp)	417.47
GC percent	61.59

Note： ORFs number: Indicates the number of genes in sample; Integrity-start: Represents the number and percentage of genes that contain only the start codon; Integrity-end: Represents the number and percentage of genes that contain only stop codons; Integrity-none: Represents the number and percentage of genes with neither start nor stop codons; Integrity-all: Represents the number and percentage of complete genes (both start and stop codons); Total Length: The total length of a gene catalogue; Average Length: Indicates the average length of genes in the gene catalogue; GC Percent: Represents the total GC content of the gene in the predicted gene catalogue.
